# Multiple Uses of Wild Edible Trees by a Nahua-Origin Community in Western Mexico

**DOI:** 10.3390/plants13233334

**Published:** 2024-11-28

**Authors:** Alana Pacheco-Flores, Rubén Ortega-Álvarez, María Guadalupe Carrillo-Galván, Manuel J. Cach-Pérez, Emanuel Ruiz-Villarreal, Alejandro Casas

**Affiliations:** 1 Instituto de Investigaciones en Ecosistemas y Sustentabilidad, Universidad Nacional Autónoma de México, Antigua Carretera a Pátzcuaro No. 8701, Morelia 58190, Michoacán, Mexico; 2 Posgrado en Ciencias Biológicas, Universidad Nacional Autónoma de México, Unidad de Posgrado, Edificio D, 1° Piso, Circuito de Posgrados, Ciudad Universitaria, Coyoacán, Ciudad de México 04510, Mexico; 3 Jardín Etnobiológico La Campana, Villa de Álvarez 28977, Colima, Mexico; lupita.carrillog@gmail.com (M.G.C.-G.); ruiz.ciages2014@gmail.com (E.R.-V.); 4 Centro de Investigación en Alimentación y Desarrollo, A.C., Centro de Estudios e Investigación en Biocultura, Agroecología, Ambiente y Salud Unidad Colima, Centro Universitario de Gestión Ambiental y Ecoparque Nogueras, Hacienda Nogueras s/n, Comala 28454, Colima, Mexico; 5 Programa Investigadoras e Investigadores Por México, Consejo Nacional de Humanidades, Ciencias y Tecnologías, Av. Insurgentes Sur 1582, Col. Crédito Constructor, Ciudad de México 03940, Mexico; 6 El Colegio de la Frontera Sur, Unidad Villahermosa, Carretera a Reforma Km. 15.5 s/n, Guineo 2da. Sección, Villahermosa 86280, Tabasco, Mexico; mcach@ecosur.mx

**Keywords:** agrobiodiversity, agroecology, ethnoecology, food sovereignty, plant management, traditional foods, tropical dry forest, Sierra de Manantlán Biological Reserve

## Abstract

Wild edible trees (WETs) play an important role in the diet of many rural communities. Therefore, research on their use and management is important to support both food sovereignty and local conservation of biocultural resources. We evaluated the different uses of WETs by the community of Zacualpan, Colima, in western Mexico, through 32 semi-structured interviews registering the species richness, plant parts consumed, and non-food uses. Additional information was collected on their management, availability, and forms of preparation. We used a cultural salience index to determine the food importance of the WETs mentioned. We registered 33 edible tree species (26 wild, 3 native crops, and 4 exotic crops) that were most commonly consumed as fruits or seeds. WETs were also used for fuel, live fences, timber, handicrafts, medicine, shade, fodder, poles, utensils, construction, tanning, soap, and paper. *Pithecellobium dulce* had the highest food salience, followed by *Spondias purpurea*, *Leucaena esculenta*, *Leucaena leucocephala*, *Enterolobium cyclocarpum*, and *Jacaratia mexicana*. Salient wild trees were collected in the wild and promoted in agroforestry systems. These trees provided food during the dry season and also had the highest number of additional uses. Promoting the biocultural value of WETs and their sustainable use can favor ecosystem conservation and local food sovereignty.

## 1. Introduction

The continuous search for and provision of food has been a major driver of plant management. Prehistoric vegetation management practices were used to increase the abundance and availability of edible annual and perennial plants, laying the foundation for plant cultivation and domestication [1,2]. These practices have persisted in traditional agricultural systems, generating a rich diversity of foods, knowledge, and skills for their use and procurement that sustain the sovereignty of many rural communities worldwide [3,4]. In recent centuries, however, human diets have become increasingly concentrated on a small number of domesticated species. As a result, 80% of the global dietary energy intake now comes from just a few crops, while wild species receive far less attention. This shift has led to a significant decline in dietary and biocultural knowledge [5].

Despite the accelerated decline in the use of wild edible plant species, they are still consumed in both industrialized and developing countries [4]. Because of their cultural and economic importance and to ensure their availability, wild and domesticated plants are managed both in situ and ex situ [6,7]. Domesticated species are those that have been genetically modified by human selection, often resulting in morphological, physiological, reproductive, and genetic divergence from wild populations [8]. However, many wild species are subject to different types of management practices that may result in intermediate or advanced degrees of domestication. Over time, wild species may be cultivated and managed in other forms that procure and enhance their maintenance in forest and agroforestry areas as non-domesticated or semi-domesticated plants [7,8].

For example, ethnobotanists have identified 251 domesticated species of native crops in Mexico and Central America [9], nearly 700 species of semi-domesticated plants, and about 7000 species obtained through gathering, including 1700 useful trees [10]. These numbers correspond to plant species used for different purposes, but food plants are outstanding. Mapes & Basurto [11] recorded nearly 2200 edible plant species occurring in Mexico, of which 542 are trees used for food [12]. Most of these are wild edible trees (WETs), which play an important role in the diet of many rural communities [4,11,13,14].

WETs provide a variety of nutritional and nutraceutical products that contribute to dietary diversification and promote health benefits [5,15]. Furthermore, WETs also serve as a vital resource in times of scarcity or famine, especially when conventional crops are unavailable or fail [16,17]. In addition, their structural characteristics and longevity make them valuable multipurpose resources that are commonly used for construction, fuel, fodder, medicine, and other purposes [18,19]. All these characteristics make WETs an important source of household income through the sale of their products in local markets and nearby urban areas [6,13]. To obtain multiple benefits, these species are often managed in wild vegetation, agroforestry systems, fallow land, and home gardens, making them an important component of agrobiodiversity [20,21,22].

In this context, WETs have cultural values, where traditional ecological knowledge (TEK) is intricately woven into local culinary practices, sentiments, spirituality, agriculture, and ecosystem management, shaping community identity and biocultural heritage [19,23,24]. These food systems also include means to communicate and transmit environmental knowledge, including information on the harvesting, processing, and sustainable use of edible plants, their seasons and production cycles, their habitats, and their use by other species [25]. Thus, the cultural importance of WETs can promote their management over time as they are passed down through generations, which can promote ecosystem regeneration and conservation [22].

WETs also serve as food resources for wildlife, such as birds and bats, which act as important dispersers and pollinators [26,27]. In addition, their presence in agroecosystems and even in urban areas promotes ecosystem connectivity, supports soil fertility, and helps prevent erosion [28,29]. These functions reinforce the importance of local knowledge and biocultural memory in protecting, promoting, conserving, and restoring biodiversity [24]. However, research on WETs in the Americas has often been relegated to general studies of useful wild plants [4,11,17,19,30,31,32] and the management and domestication of edible trees [6,9,33,34,35]. Given the diversity of WETs, research in understudied regions focusing on their multiple uses, nutritional and nutraceutical properties, economic importance, and ecology is still needed. 

Our study aimed to (1) evaluate the cultural importance of WETs in meeting human needs, analyzing their use as food sources, including the plant parts consumed, their availability, preparation forms, and additional non-food uses; (2) examine the relation of these uses with actions to procure, conserve and promote the abundance of trees by people of the community of Zacualpan, Colima, in western Mexico; and (3) evaluate the cultural values of forest components and the importance of maintaining such values for promoting biodiversity conservation programs. The area where the study was conducted is part of the Central–Western Mexico Biocultural Corridor and the Nevado de Colima-Sierra de Manantlán Biological Corridor, which is shared by the states of Colima and Jalisco. It is, therefore, an area of high priority for biodiversity conservation and biocultural importance, as indigenous people have been present in the area for hundreds of years. However, despite this importance, biological inventories and ethnobotanical research remain limited, especially in Colima. 

Zacualpan is one of the last two indigenous communities in the state of Colima with communal land tenure, recognized for its efforts to preserve local traditions and common goods. They faced the threat of an extractive mining project and several problems associated with the provision of water to urban areas of the city of Colima [36]. Our research in the area has recognized local initiatives from the community to document their culinary traditions, and more recently, our research group has conducted studies on Zacualpan’s agroforestry systems [22,37]. Therefore, documenting the cultural motivations for conserving forest elements in agroforestry systems is particularly important for promoting conservation programs. In-depth research on these issues is therefore needed. In this context, our study contributes to a deeper understanding of indigenous uses of tree resources and highlights the importance of WETs for human well-being, food sovereignty, and ecosystem conservation.

## 2. Results

### 2.1. Species Richness and Composition of WETs

We recorded 33 species of edible trees belonging to 18 botanical families, based on the local classification of trees by the Zacualpan people, which included trees, an arborescent cactus (*Stenocereus queretaroensis* (F.A.C. Weber) Buxb.), and a bamboo (*Otatea acuminata* Munro (C.E. Calderón & Soderstr.). The highest number of species was found in the families Fabaceae (six species), Anacardiaceae (four species), and Annonaceae (three species). The local population consumed 26 WETs, including six endemic and one threatened species. Domesticated trees included three native and four introduced species (Table S1).

**Figure 1:**
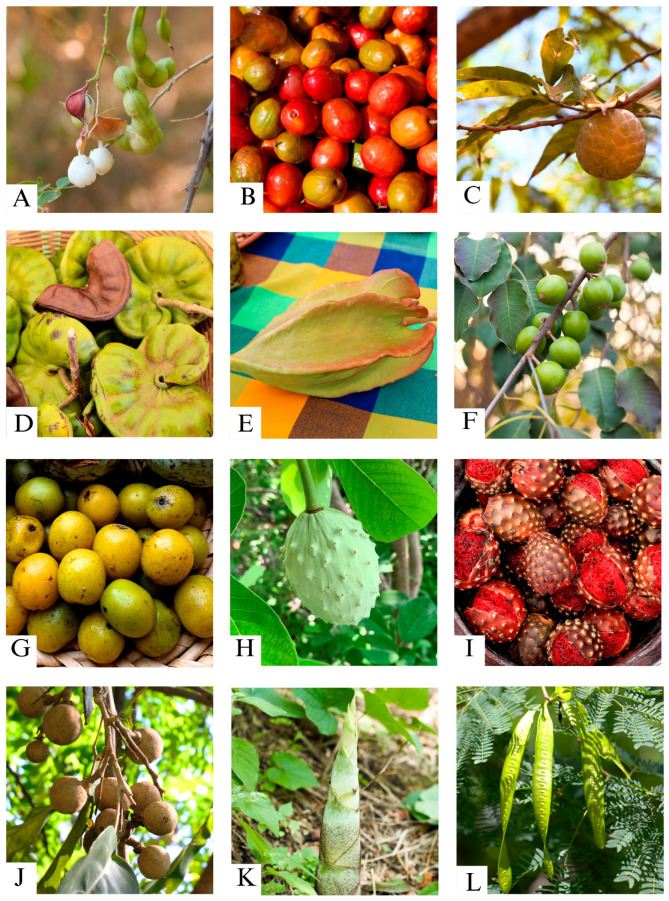
Wild edible trees consumed by the community of Zacualpan: (**A**) *Pithecellobium dulce* (guamúchil) fruits; (**B**) *Spondias purpurea* (ciruelo) fruits; (**C**) *Annona reticulata* (anona) fruits; (**D**) *Enterolobium cyclocarpum* (parota) seed pods; (**E**) *Jacaratia mexicana* (bonete) fruits; (**F**) *Sideroxylon capiri* (capire) fruits; (**G**) *Cyrtocarpa procera* (chupalcojote) fruits; (**H**) *Annona macroprophyllata* (ilama) fruits; (**I**) *Stenocereus queretaroensis* (pitayo) fruits; (**J**) *Morisonia americana* (zapote barranqueño) fruits; (**K**) *Otatea acuminata* (otate) sprouts; and (**L**) *Leucaena leucocephala* (guaje verde) unripe seed pods.

Guamúchil (*Pithecellobium dulce* [Roxb.] Benth.) [frequency = 29] had the highest food salience among edible trees, followed by ciruelo (*Spondias purpurea* L.) [frequency = 16], guaje rojo (*Leucaena esculenta* [DC.] Benth.) [frequency = 15], guaje verde (*Leucaena leucocephala* (Lam.) de Wit) [frequency = 13], parota (*Enterolobium cyclocarpum* [Jacq.] Griseb.) [frequency = 12], and bonete (*Jacaratia mexicana* A. DC.) [frequency = 11]. Four of the most salient species belong to the Fabaceae family (guamúchil, guaje rojo, guaje verde, and parota) (Figure 1; Table 1). There were no statistical differences between women and men related to the knowledge of edible trees (χ^2^ = 28.1, df = 32, *P* = 0.6). According to our estimates, 39 edible tree species could be detected (lower confidence limit: 31.1; upper confidence limit: 46.1) if more people were included in the survey (64 respondents) (Figure S1; Table S2).

### 2.2. Parts of the Plant Consumed

**Figure 2:**
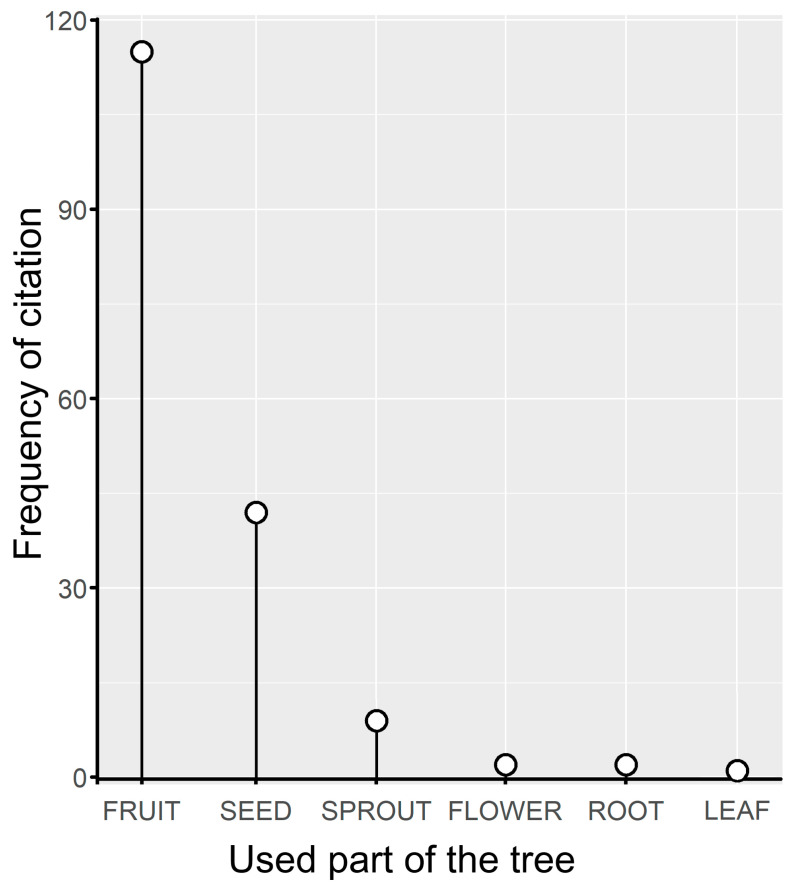
Tree parts consumed by people of the community of Zacualpan, Colima, Mexico.

Fruits, seeds, sprouts, flowers, roots, and leaves were consumed by the community (Figure 2). However, the frequency of citation counts was not evenly distributed among the edible parts of the trees (χ^2^ = 358.09, df = 5, *P* < 0.001), as fruits and seeds were locally preferred for consumption. Although 28 tree species were used to obtain fruits, guamúchil (frequency = 29; 25.2%), ciruelo (frequency = 16; 13.9%), and bonete (frequency = 11; 9.5%) were the main sources of this resource. Only guajes (three species; frequency = 29; 69%) and parota (frequency = 12; 31%) were valued for their seeds.

**Figure 3:**
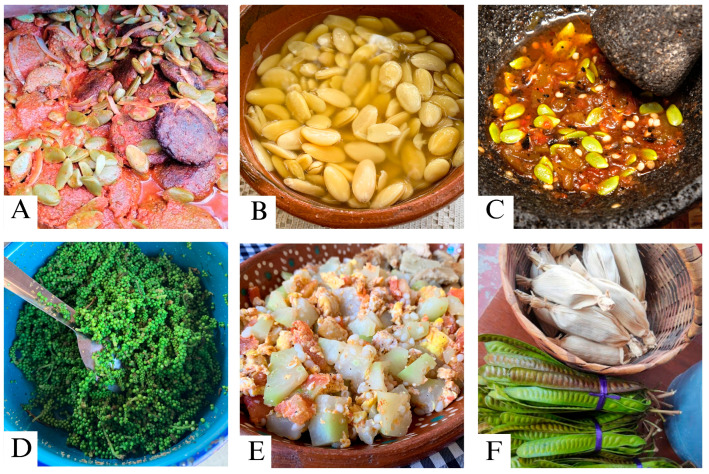
Preparation forms of some WETs recorded in the community of Zacualpan: *E. cyclocarpum* (parota) seeds as (**A**) tortitas, (**B**) soup; (**C**) chili sauce with guaje verde (*L. leucocephala)* unripe seeds; (**D**) *L. macrantha* (embiona) flower sprouts cooked for tacos; (**E**) scrambled eggs with *J. mexicana* (bonete) unripe fruits; (**F**) unripe seed pods of guaje verde and *S. purpurea* (ciruelo) fruits preserves as tamalitos.

The availability of edible parts of the WETs was variable throughout the year and helped to supplement people’s diets. The most salient species (guamúchil, ciruelo, guaje rojo, guaje verde, parota, and bonete) and 10 other species were consumed during the dry season (i.e., Nov–Jun) (Table 1). These species also had different ways of consumption and incorporation into traditional dishes, including cooked, dried, fresh, flavored water, roasted, and toasted (Figure 3, Table S3). All fruits were consumed fresh. In addition, the unripe fruits of the bonete were used as a vegetable. Ciruelo showed the most diverse forms of preparation, including fresh, flavored water, preserves, alcoholic beverages, chili sauce, stew, and dried. The leafy sprout of the guajes was consumed fresh while the otate sprout was roasted. Only the flower sprout of the embiona tree was consumed. Ciruelo fruits and mature parota seeds were dried for later consumption, as were the unripe bonete fruits preserved with vinegar.

### 2.3. Multiple Uses of WETs

**Figure 4:**
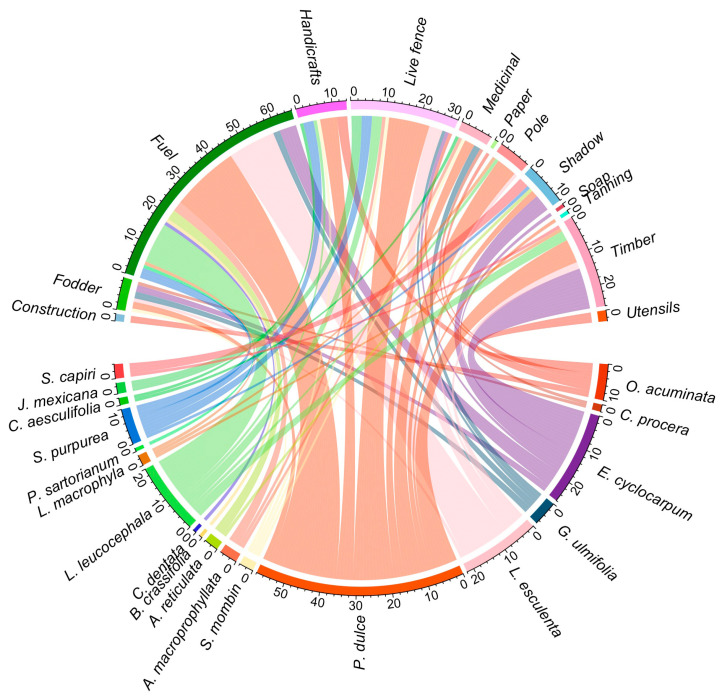
Chord diagram representing the other uses of wild edible tree species by the community of Zacualpan, Colima, Mexico.

Edible trees were also used by the community for 13 different purposes: fuel, live fence, timber, raw material for handicrafts, medicine, shade, fodder, poles, utensils, construction materials, tanning material, soap, and paper (Figure 4). Frequencies varied across uses (χ2 = 268.69, df = 12, *P* < 0.001). Fuel (frequency = 64; 35.3%), live fence (frequency = 29; 16%), and timber (frequency = 27; 14.9%) were the most common non-feed uses of edible trees. Guamúchil had the highest number of other uses among the trees (9), followed by parota (6), guaje verde (6), guaje rojo (5), ciruelo (5), and otate (5). All the parts of these trees were used by the community to meet many of their household needs, such as medicine, fuel, and utensils, as well as to support their work, such as fodder, live fences, poles, and shade (Table S1).

### 2.4. Management of WETs

Edible trees were managed in different ways within home gardens, agroforestry systems, fallow land, and forest areas: (1) gathered or collected in the wild; (2) tolerated or left standing in the areas that were cleared for various purposes; (3) providing special protection against damage from fire, herbivores, wind, and other factors, or by providing to them light or shade for some individual plants; (4) promoting their abundance by sowing their seeds, planting vegetative propagules or transplanting young plants; and (5) cropped (planting domesticated plants that are subject to human selection). 

The most salient species (guamúchil, ciruelo, guaje rojo, guaje verde, parota, and bonete) had economic importance, as did anona (*A. reticulata* L.), chupalcojote (*C. procera* Kunth), guayabillo (*P. sartorianum* (O. Berg) Nied.), ilama (*A. macroprophyllata* Donn. Sm.), nance (*B. crassifolia* (L.) Kunth), and pitayo (*S. queretaroensis* (F.A.C. Weber) Buxb.), which were commonly sold in local markets. These species were specifically protected and promoted in home gardens and through agroforestry practices, such as live fences, isolated trees, and vegetation patches. Conversely, species such as ahuilote (*V. mollis* Kunth), granjeno (*C. iguanaea* (Jacq.) Sarg.), and zapotillo negro (*Diospyros* sp. L.), were tolerated, and their fruits were typically consumed when encountered in the wild. Domesticated species were cropped and included avocado (*Persea americana* Mill.), guanábana (*Annona muricata* L.), guava (*Psidium guajava* L.), lemon (*Citrus aurantifolia* (Christm) Swingle), mango (*Mangifera indica* L.), papaya (*Carica papaya* L.), and tamarind (*Tamarindus indica* L.).

## 3. Discussion

### 3.1. Species Richness and Composition of WETs 

Our results show that the use of wild edible tree species (26) recorded in Zacualpan is high (79%) compared to domesticated species (21%); this may reflect the importance of TEK and local adaptations to drought in the use and management of the tropical dry forest (TDF). Sixteen WET species were consumed during the dry season, with the most salient species (guamúchil, ciruelo, guaje rojo, guaje verde, parota, and bonete) providing abundant fruits and seeds. Furthermore, the use and management of TDF in western Mexico, dating back to 9000 years BP, has been closely associated with the domestication and diversification of milpa species (i.e., maize, squash, beans), but also with the management of edible trees such as ciruelo and guaje rojo [2]. This highlights WETs as vital ancestral resources with significant implications for human culture, health, ecosystem management, and conservation.

The Fabaceae family had the highest number of edible species and four of the most salient species (guamúchil, guaje rojo, guaje verde, parota). This family is particularly abundant in tropical dry forests due to its various adaptations to drought and resource limitation, allowing many species to be established in disturbed areas such as cultivated fields and fallow land [38]. Such is the importance that Fabaceae species are widely used for medicinal purposes in Mexico, followed by species used for animal feed, material resources, environmental modification, and food additives [39]. In addition, these species are rich in protein, making them a valuable food source not only for humans but also for livestock [40].

The most salient edible species was guamúchil, which was also valued for the income generated by the fruit trade. Other economically important tree products were commonly sold in local markets such as anona, bonete, ciruelo, chupalcojote, guajes, guayabilla, ilama, nance, parota, and pitaya. In Zacualpan, these trees are found managed in nearby forests, home gardens, and various agroforestry areas, often undergoing several cycles of fallow and cultivation [22]. For these reasons, some of these species can be classified as wild or semi-domesticated cultivated plants. In the case of bonete, studies have shown an incipient domestication syndrome in cultivars from central and northwestern Mexico [41]. In the case of ciruelo, this tree is particularly important in the diet of the Zacualpan people, as 11 varieties of this fruit have been reported [37]. In fact, western Mexico is part of its Mesoamerican domestication range, where it coexists with wild populations [35].

### 3.2. Plant Parts Consumed

Studies of WETs conducted in Africa have shown similar patterns of use, with fruits being the most important component of edible trees, followed by seeds, roots, and leaves [16,42]. In Zacualpan, fruits were the most consumed part of the trees by the community, followed by seeds. The fruits of the ciruelo and bonete trees were particularly favored, possibly because of their abundance, versatility in preparation forms, ease of clonal propagation, and forms of preservation. Different products complement each other due to their seasonal availability, and the storage of certain items increases food security, especially during the dry season [17]. For example, ciruelo fruit, which spoils quickly, is often dried and processed into various products that can be stored and consumed throughout the year. Meanwhile, parota seeds were preferred to be cooked unripe, although dried seeds are also consumed and can be stored for later use. In fact, some forms of preparation of these trees in Zacualpan, such as sun-drying ciruelo fruits for later rehydration to make *tamales* or toasting and grinding parota seeds, are considered pre-Ceramic archaic components of the region’s human diet [43].

However, many WETs species were consumed only occasionally. Flower sprouts and leaf sprouts were the least consumed tree parts, mainly due to their quantity and short period of edible availability. Given the cultural importance of many species, their promotion by the Zacualpan people is common, but the consumption of fruits and seeds of endangered (e.g., capire) and endemic species that are less promoted (e.g., ahuilote) needs to be evaluated in future studies from a sustainability perspective.

### 3.3. Multiple Uses of WETs

In Zacualpan, the main non-food uses of WETs were for fuelwood, fencing, and timber, which have been reported as common additional uses of WETs in other parts of the world [14,18,44]. Fuelwood collection is common in rural communities to meet their heating needs and is largely determined by the proximity and accessibility of trees. Typically, a wide variety of trees are used for fuelwood, many of which are also used for edible purposes [45]. Live fences are mainly used in tropical areas to demarcate and protect agricultural plots and home gardens, but they also serve as multifunctional systems that provide fodder, fuel, timber, food, medicine, and wind barriers and help prevent soil erosion [46]. In Zacualpan, the most salient species, the guamúchil tree, was particularly valued, with nine non-food uses in addition to its role as a food source, and was commonly managed in live fences and home gardens as previously reported [22,27].

Other studies have shown that, in addition to providing food, edible trees serve multiple purposes that support the livelihoods of many communities worldwide [13,14,18,19]. However, many of these species are often exploited more for their non-food uses than for their nutritional value. Overharvesting of WETs for fuelwood, medicine, fencing, construction, and fodder can exacerbate the degradation of certain species [42]. Selective harvesting of forest resources can lead to localized declines in species, basal area, and density, favoring those more resilient to extraction [47]. In addition, livestock browsing contributes to soil compaction, the loss of forest understory, and the inhibition of new plant recruitment [48].

### 3.4. Ecological Implications and Challenges in the Management of WETs

Some of the ecological impacts associated with the use of trees and land use change can be mitigated through local sustainable practices. For example, in Mexico, WETs are often protected and promoted both in situ and in cultivated stands to increase their availability and favor desirable phenotypes [6]. Similarly, in other countries these forms of interactions have been recorded. For instance, in Ethiopia, WETs have been managed by deliberately leaving trees on farmland and occasionally planting important species in home gardens, reflecting common shared cultural practices for managing edible trees [42]. These agroforestry practices can help reduce pressure on conservation and protected areas [49]. In addition, systems such as home gardens and practices such as live fences act as in situ gene banks that serve as reservoirs of genetic diversity for cultivated species, especially when native populations of their wild ancestors are declining [35]. Furthermore, these species also provide food resources for wildlife and act as stepping stones that enhance ecological connectivity among populations, communities, and processes [27,28,50]. 

Despite their ecological importance, WETs and other wild foods are often stigmatized and associated with poverty [13,44]. In addition, several challenges limit their promotion and commercialization, such as the lack of experience, low profitability, limited availability, and the lack of recognition of the market value of these species [13]. To address these challenges, the biocultural promotion and sustainable cultivation of WETs can serve as a key strategy. Local efforts to reclaim the importance of wild foods as part of the biocultural heritage have emerged, led by women through traditional cuisine movements. In Zacualpan, initiatives such as Grupo Xolocuahuitl, the Indigenous Council of Zacualpan, and their collaboration with other collectives such as Mujeres del Fuego, Frente en Defensa del Maíz de Colima, and Jardín Etnobiológico La Campana are examples of these efforts [37,51,52,53]. Thus, by incorporating WETs into cultural and agroforestry programs that emphasize their nutritional benefits, economic potential, and role in conserving TEK, WETs can contribute significantly to local food sovereignty and ecosystem conservation.

## 4. Materials and Methods

**Figure 5:**
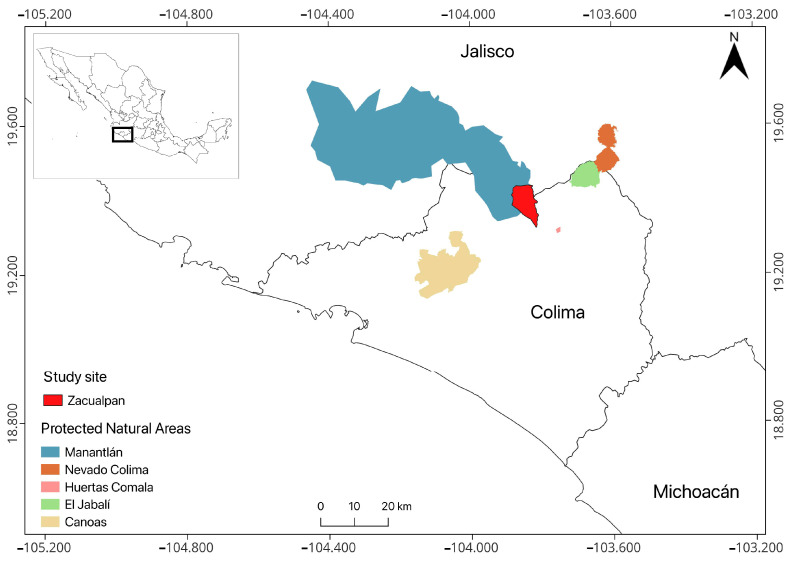
Localization of the community of Zacualpan, Colima, Mexico.

Our research was conducted in the Nahua-origin community of Zacualpan, municipality of Comala, Colima, Mexico. The area is part of the Nevado de Colima-Sierra de Manantlán Biological Corridor and the Central–Western Mexico Biocultural Corridor (Figure 5). The main local productive activity is agriculture, which consists mainly of traditional rainfed agriculture and livestock ranching (i.e., goats and cows). Trade, livestock, and river fishing are other important economic activities in the community. Tropical dry forest is the main type of vegetation, followed by riparian forest. About 2000 people live in the territory, which consists of a small urban settlement (~30 ha), a matrix of agricultural fields, forest, fallow land, springs, and the Armería River. According to the Köppen climate classification system modified by García [54], the climate of Comala is semi-warm subhumid with summer rains [(A)C(wo)(w)], with an average annual temperature of 21.5 °C, a total annual precipitation of 761 mm, rainfall from June to November, and a pronounced dry season from November to June. Zacualpan is one of the last indigenous communities in the state of Colima with communal land tenure; it is recognized for its efforts to preserve its traditions and biocultural heritage. 

In July and August of 2022, we conducted 32 random semi-structured interviews to learn about the trees used for food by the community of Zacualpan. We focused our survey on farmers because they have extensive knowledge of the biocultural edible resources that are used by the community. People interviewed also reported other occupations, including trader, mason, artisan, teacher, and housekeeper. We included an equal number of men and women in the survey (16 participants of each gender), ranging in age from 30 to 90 years old (median = 53 years). During the interviews, we asked the people to name the tree species that provide food for the community, as well as the parts of the plants that are consumed. We listed the species that were mentioned by the interviewees and then asked about the non-food uses that the local people gave to each of them. Again, we recorded the parts of the trees that were used by the local people.

For trees, we used the local classification of the Zacualpan people, which included two perennial species: the columnar cactus (*Stenocereus queretaroensis* (F.A.C. Weber) Buxb.) and a bamboo (*Otatea acuminata* Munro (C.E. Calderón & Soderstr.). Botanical samples for species identification of WETs were collected, preparing voucher specimens and photographic records and during field surveys in collaboration with local botanists, as described in Pacheco-Flores et al. [22]. To determine the food importance of the trees that were mentioned by the community members, we used a free list method to calculate a cultural salience index [55]. Specifically, we used the B’score index because it allows the comparison between the elements of the list regardless of its length or the number of respondents since it varies between 0 and 1 [56]. The B’score was calculated by combining the frequency of mention and the rank of citation of each item in the list. We used the open-source software FLARES v 1.0 to calculate the index [57].

We estimated the number of tree species that could be used as food sources by the community. Estimates were made using the “iNext” package [58] in R (R Core Team 2020). Each respondent was considered as a sampling unit for our analysis (*n* = 32). The frequency of occurrence of trees reported by respondents was used to compute sample-size-based rarefaction (interpolated estimation) and extrapolation (predictive estimation) sampling curves for edible tree species richness. We compared (i) the consumption of different parts of the plants and (ii) their non-food uses with a Chi-Squared Test of Homogeneity. Finally, we used additional interviews, field notes, direct and participant observation, and local phenology data of WETs to learn about tree management, temporal availability, and the preparation forms of these foods.

## 5. Conclusions

The knowledge of wild edible plants and their multiple uses is an invaluable heritage that is being steadily eroded by shifts in food systems and the globalization of diets, which has led to a significant decline in health and TEK. The continuing loss of these resources highlights the urgent need for expanded research and social initiatives to safeguard this knowledge for future generations and to strengthen food sovereignty, security, and sustainability.

Wild edible trees play an important role in the livelihoods of the community of Zacualpan, not only for food but also to provide materials for their daily life and work activities. The use of 26 native species, especially 16 that were consumed during the dry season, reflects the importance of TEK and local adaptations to drought in the use and management of the tropical dry forest. This highlights WETs as ancestral resources with significant implications for human culture, health, and ecosystem management.

Strengthening fair market incentives through community-based initiatives, education, and policy support can help to change perceptions, making WETs a valuable biocultural resource rather than a symbol of scarcity or poverty. Therefore, the culinary promotion, cultivation, and sustainable management of WETs in agroforestry systems can be a key strategy to support both local food sovereignty and biodiversity conservation. Ultimately, these efforts can promote the dual objectives of supporting human well-being and conserving ecosystems, which are in line with the global goals of sustainable development. 

## Figures and Tables

**Table 1:** WETs and their edible parts, harvest season, forms of preparation, and other uses recorded in the community of Zacualpan, Colima, Mexico. Tree species: (E) = endemic; (T) = threatened. RFM = relative frequency of mention, B’score = food salience. Harvest: DS = dry season; RS = rainy season; D&RS = dry and rainy season. Management types: (G) = gathering, (T) = tolerance, (SP) = special protection, and (P) = promotion. See descriptions of these management types in the main text.

Family/Tree Species/Common Name	RFM	B’ Score	Management	Edible Part	Harvest	Preparation Forms	Other Uses of WETs
FABACEAE *Pithecellobium dulce* (Roxb.) Benth. (Manila tamarind; guamúchil)	0.91	0.71	G, T, SP, P	Fruit	Jan–May (DS)	Fresh, cooked with maize dough	Fuel, medicinal, live fence, fodder, pole, tanning, timber, shade, handicrafts
ANACARDIACEAE *Spondias purpurea* L. (Hog plum; ciruelo)	0.50	0.37	G, T, SP, P	Fruit	Mar–May (DS)	Fresh, flavored water, preserves, alcoholic beverages, chili sauce, unripe fruits in stew with beans, dried fruits are consumed all year	Fuel, handicrafts, fodder, shade, live fences
FABACEAE *Leucaena esculenta* (DC.) Benth. (E) (Guaje Rojo o Colorado)	0.47	0.40	G, T, SP, P	Seed, leaf sprout	Dec–Feb (DS)	Fresh, grounded in chili sauce. Fresh leaf sprout	Fuel, fodder, live fences, timber, pole
*Leucaena leucocephala* (Lam.) de Wit (White leadtree; guaje verde)	0.41	0.29	G, T, SP, P	Seed, leaf sprout	Jan, Jun, Sep (D&RS)	Fresh, ground in chili sauce. Fresh leaf sprout	Fuel, live fences, timber, pole, handicrafts, shade
*Enterolobium cyclocarpum* (Jacq.) Griseb. (Guanacaste tree; parota)	0.38	0.25	G, T, SP, P	Seed, germinated seed	Mar–Jun (DS)	Cooked unripe seeds in soups and dishes, dry seeds preserved toasted as popcorn, germinated seeds cooked	Timber, shade, fodder, soap, fuel, live fences
CARICACEAE *Jacaratia mexicana* A. DC. (Bonete)	0.34	0.24	G, T, SP, P	Fruit	Nov–Apr (DS)	Mature fruit eaten fresh; unripe fruit eaten fresh and cooked as a vegetable. Unripe fruit preserved in vinegar	Live fence
ANNONACEAE *Annona macroprophyllata* Donn. Sm. (Ilama)	0.25	0.18	G, T, SP, P	Fruit	Sep–Nov (RS)	Fresh	Medicinal, fuel, live fences
*Annona reticulata* L. (Custard apple; Anona)	0.22	0.16	G, T, SP, P	Fruit	Mar–Nov (D&RS)	Fresh	Shade, fuel
ANACARDIACEAE *Cyrtocarpa procera* Kunth (E) (Chupandia; chupalcojote)	0.16	0.11	G, T, SP, P	Fruit	Sep–Oct (RS)	Fresh	Fodder, medicinal
*Spondias mombin* L. (Plum; ciruelo de monte, ciruelo de aguas)	0.16	0.09	G, T, SP	Fruit	Jul–Nov (RS)	Fresh, flavored water	Handicrafts, medicinal
POACEAE *Otatea acuminata* (Munro) C.E. Calderón & Soderstr. (E) (Mexican weeping bamboo; otate)	0.16	0.08	G, T, SP, P	Sprout	Jul–Sep (RS)	Grilled	Handicrafts, utensils, live fences, construction, paper
MALVACEAE *Guazuma ulmifolia* Lam. (Bastard cedar; guásima)	0.09	0.05	G, T, SP, P	Fruit	Jan–Mar (DS)	Fresh and dried fruits as candy	Fodder, medicinal, fuel, live fences
MALPIGHIACEAE *Byrsonima crassifolia* (L.) Kunth (Nance)	0.06	0.03	G, T, SP, P	Fruit	May–Sep (D&RS)	Fresh, flavored water	Fuel
CACTACEAE *Stenocereus queretaroensis* (F.A.C. Weber) Buxb. (E) (pitayo)	0.06	0.03	G, T, SP, P	Fruit	Apr–May (DS)	Fresh	
CORDIACEAE *Cordia dentata* Poir. (Tambora)	0.06	0.03	G, T	Fruit	Apr–Jul (D&RS)	Fresh	Fuel
SAPOTACEAE *Sideroxylon capiri* (A. DC.) Pittier (T) (Capire)	0.06	0.03	G, T, SP	Fruit	Mar–Jun (DS)	Fresh, cooked	Timber, shade
PETIVERACEAE *Ledenbergia macrantha* Standl. (Embiona)	0.06	0.02	G, T, SP, P	Flower sprout, leaf sprout	Jan–Mar (DS)	Cooked flower sprouts, fresh leaf sprouts	Live fences
MALVACEAE *Ceiba aesculifolia* (Kunth) Britten & Baker f. (Pochote)	0.06	0.02	G, T	Fruit, root	Jan–Mar (DS)	Fresh	Medicinal, handicrafts
FABACEAE *Leucaena macrophyla* Benth. (E) (Guaje de Hoja Redonda)	0.03	0.02	G, T	Seed, leaf sprout	Jul–Sep (RS)	Fresh, ground in chili sauce. Fresh leaf sprout	Fuel, pole, timber
MYRTACEAE *Psidium sartorianum* (O. Berg) Nied. (Little guava; guayabillo)	0.03	0.02	G, T, SP, P	Fruit, leaves	Dec–Feb, Jul–Sep (D&RS)	Fresh, flavored water	Fuel
CAPPARACEAE *Morisonia americana* L. (Zapote Barranqueño)	0.03	0.02	G, T, P	Fruit	Nov–Dec (DS)	Fresh	
CANNABACEAE *Celtis iguanaea* (Jacq.) Sarg. (Iguana hackberry; granjeno)	0.03	0.01	G	Fruit	Sep–Nov (RS)	Fresh	
SAPOTACEAE *Pouteria campechiana* (Kunth) Baehni (Zapote calentura)	0.03	0	G, T, SP	Fruit	Aug–Sep (RS)	Fresh	
RUTACEAE *Casimiroa edulis* La Llave (White sapote; zapote dormilón)	0.03	0	G, T, SP	Fruit	Jun–Aug (D&R)	Fresh	
EBENACEAE *Diospyros* sp. L. (Zapotillo negro)	0.03	0	G, T	Fruit	Dec (DS)	Fresh	
LAMIACEAE *Vitex mollis* Kunth (E) (Ahuilote)	0.03	0	G, T	Fruit	Apr–Oct (D&RS)	Fresh	

## Data Availability

Data supporting the reported results and datasets generated during the study can be found in the Supplementary Information included with this manuscript.

## Supplementary Materials

The following supporting information can be downloaded at https://www.mdpi.com/article/10.3390/plants13233334/s1, Table S1: Trees that were used for food by the community of Zacualpan, Colima, Mexico; Figure S1: Estimated richness of tree species that were used for food by the community of Zacualpan, Colima, Mexico; Table S2: Estimations of the number of tree species used by the community of Zacualpan for food purposes. Table S3. Preparation methods for some WETs.
