# Supplementary material for: Multiple Uses of Wild Edible Trees by a Nahua-Origin Community in Western Mexico
Source: Plants (Basel). 2024 Nov 28;13(23):3334. doi: 10.3390/plants13233334 (PMC11644277; doi:10.3390/plants13233334)
Supplement: Supplementary file 1 [file plants-13-03334-s001.zip › Pacheco-Flores et al._Supplementary information_Figure S1_TableS2.pdf]

plants

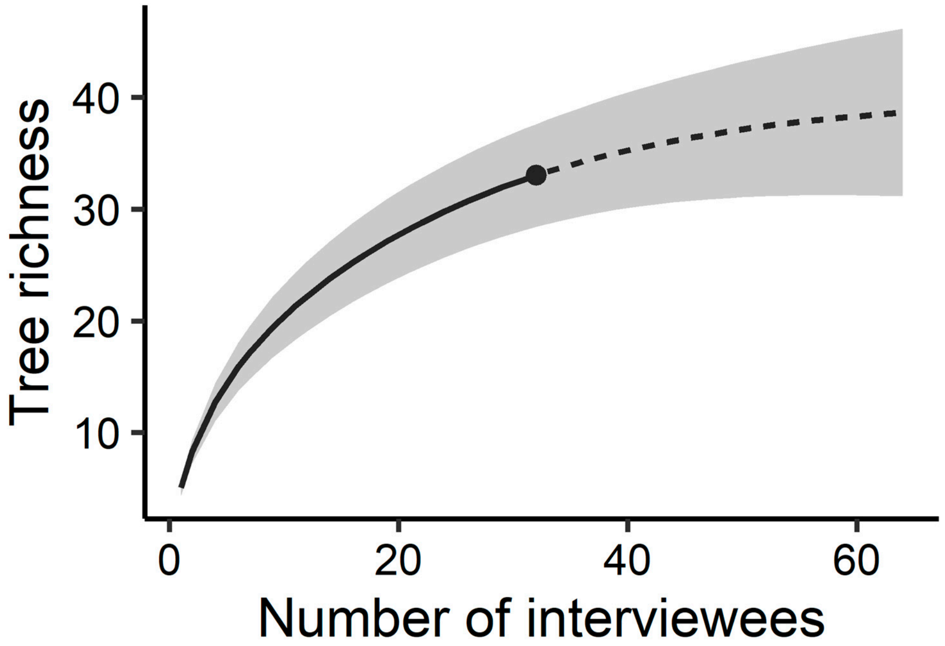

Figure S1. Estimated richness of tree species that were used for food by the community of Zacualpan, Colima, Mexico.

Table S2. Estimations of the number of tree species used by the community of Zacualpan for food purposes. Sample size (number of interviewees), method, estimated species richness (^qD), and 95% lower (^qD. LCL) and upper (^qD. UCL) confidence limits of estimations are depicted.

Sample size	Method	^qD	^qD. LCL	^qD. UCL
1	interpolated	5.1	4.4	5.8
2	interpolated	8.3	7.2	9.4
4	interpolated	12.8	11.1	14.4
6	interpolated	15.9	13.7	18.0
7	interpolated	17.2	14.8	19.5
9	interpolated	19.4	16.7	22.1
11	interpolated	21.3	18.3	24.3
12	interpolated	22.2	19.1	25.3
14	interpolated	23.8	20.4	27.1
16	interpolated	25.2	21.7	28.8
17	interpolated	25.9	22.3	29.5
19	interpolated	27.1	23.3	30.9
21	interpolated	28.2	24.3	32.2
22	interpolated	28.8	24.8	32.8
24	interpolated	29.8	25.7	33.9
26	interpolated	30.7	26.5	34.9
27	interpolated	31.1	26.8	35.4
29	interpolated	31.9	27.5	36.3

31	interpolated	32.7	28.1	37.2
32	observed	33.0	28.4	37.6
33	extrapolated	33.3	28.7	38.0
34	extrapolated	33.6	28.9	38.4
36	extrapolated	34.2	29.4	39.1
37	extrapolated	34.5	29.6	39.4
39	extrapolated	35.0	29.9	40.1
41	extrapolated	35.5	30.2	40.7
42	extrapolated	35.7	30.4	41.0
44	extrapolated	36.1	30.6	41.6
46	extrapolated	36.5	30.8	42.2
47	extrapolated	36.6	30.9	42.4
49	extrapolated	37.0	31.0	42.9
50	extrapolated	37.1	31.1	43.2
52	extrapolated	37.4	31.1	43.7
54	extrapolated	37.7	31.2	44.1
55	extrapolated	37.8	31.2	44.3
57	extrapolated	38.0	31.2	44.8
59	extrapolated	38.2	31.2	45.2
60	extrapolated	38.3	31.2	45.4
62	extrapolated	38.5	31.2	45.8
64	extrapolated	38.6	31.1	46.1
