# Supplementary material for: Multiple Uses of Wild Edible Trees by a Nahua-Origin Community in Western Mexico
Source: Plants (Basel). 2024 Nov 28;13(23):3334. doi: 10.3390/plants13233334 (PMC11644277; doi:10.3390/plants13233334)
Supplement: Supplementary file 1 [file plants-13-03334-s001.zip › Pacheco-Flores et al._Supplementary information_Table S1.pdf]

plants MDPI

Table S1. Trees that were used for food by the community of Zacualpan, Colima, Mexico. We depict the botanic family, scientific name of the species, the relative frequency of mention by interviewees (RFM), the food salience of species (B'score), the parts of the tree that were consumed, non-food uses, and the parts that were utilized. Species are arranged in descending order according to their food salience. If English and local names did not vary, just one is shown. E= endemic; T= threatened (NOM059-SEMARNAT-2010). PR= Photographic record.

Family/Tree species/ (English name; local name)	RFM	B' score	Consumed tree part	Non-food uses	Tree part used	Collection number/ PR
FABACEAE						
Pithecellobium dulce (Roxb.) Benth. (Manila tamarind; Guamúchil)	0.91	0.71	Fruit	Fuel	Stem, branches	393
				Medicinal	Fruit, bark	
				Live fence	Whole tree	
				Fodder	Fruit	
				Pole	Stem	
				Tanning	ND	
				Timber	Stem	
				Shade	Whole tree	
				Handicrafts	Stem	
ANACARDIACEAE						
Spondias purpurea L. (Hog plum; Ciruelo)	0.50	0.37	Fruit	Fuel	Stem, branches	154
				Handicrafts	Stem	
				Fodder	Fruit	
				Shade	Whole tree	
				Live fence	Whole tree	
FABACEAE						
Leucaena esculenta (DC.) Benth. (E) (Guaje Rojo)	0.47	0.40	Seed, leaf sprout	Fuel	Stem, branches	394
				Fodder	Seed pod	
				Live fence	Whole tree	
				Timber	Stem	
				Pole	Stem	
FABACEAE						
Leucaena leucocephala (Lam.) de Wit (White leadtree; Guaje Verde)	0.41	0.29	Seed, leaf sprout	Fuel	Stem, branches	377
				Live fence	Whole tree	
				Timber	Stem	

				Pole	Stem	
				Handicrafts	Stem	
				Shade	Whole tree	
FABACEAE						
Enterolobium cyclocarpum (Jacq.) Griseb. (Guanacaste tree; Parota)	0.38	0.25	Seed, germinated bean	Timber	Stem	PR
				Shade	Whole tree	
				Fodder	Seed pod, seed	
				Soap	Fruit	
				Fuel	Stem, branches	
				Live fence	Whole tree	
CARICACEAE						
Jacaratia mexicana A. DC. (Bonete)	0.34	0.24	Fruit	Live fence	Whole tree	183
ANNONACEAE						
Annona macroprophyllata Donn. Sm. (Ilama)	0.25	0.18	Fruit	Medicinal	Fruit	273
				Fuel	Stem, branches	
				Live fence	Whole tree	
Annona reticulata L. (Custard apple; Anona)	0.22	0.16	Fruit	Shade	Whole tree	324
				Fuel	Stem, branches	
ANACARDIACEAE						
Cyrtocarpa procera Kunth (E) (Chupandia; Chupalcojote)	0.16	0.11	Fruit	Fodder	Fruit	310
				Medicinal	Fruit	
Spondias mombin L. (Plum; Ciruelo de monte, Ciruelo de aguas)	0.16	0.09	Fruit	Handicrafts	Stem	392
				Medicinal	Fruit	
POACEAE						
Otatea acuminata (Munro) C.E. Calderón & Soderstr. (E) (Mexican weeping bamboo; Otate)	0.16	0.08	Sprout	Handicrafts	Stem	225
				Utensils	Stem	
				Live fence	Whole tree	
				Construction	Stem	
				Paper	ND	
MYRTACEAE						
Psidium guajava L. (Guava; Guayabo)	0.13	0.08	Fruit	Timber	Stem	
				Fuel	Stem, branches	

				Medicinal	ND	
FABACEAE						
Tamarindus indica L. (Tamarind; Tamarindo)	0.09	0.05	Fruit	Fuel	Stem, branches	
MALVACEAE						
Guazuma ulmifolia Lam. (Bastard cedar; Guásima)	0.09	0.05	Fruit	Fodder<br>Medicinal<br>Fuel<br>Live fence	Fruit<br>Fruit<br>Stem, branches<br>Whole tree	287
MALPIGHIACEAE						
Byrsonima crassifolia (L.) Kunth (Nance)	0.06	0.03	Fruit	Fuel	Stem, branches	PR
CACTACEAE						
Stenocereus queretaroensis (F.A.C. Weber) Buxb. (E) (Pitayo)	0.06	0.03	Fruit			PR
CORDIACEAE						
Cordia dentata Poir. (Tambora)	0.06	0.03	Fruit	Fuel	Stem, branches	368
SAPOTACEAE						
Sideroxylon capiri (A. DC.) Pittier (T) (Capire)	0.06	0.03	Fruit	Timber<br>Shade	Stem<br>Whole tree	319
ANNONACEAE						
Annona muricata L. (Guanábana)	0.06	0.03	Fruit			
ANACARDIACEAE						
Mangifera indica L. (Mango)	0.06	0.03	Fruit			
PETIVERACEAE						
Ledenbergia macrantha Standl. (Embiona)	0.06	0.02	Flower sprout, leaf sprout	Live fence	Whole tree	372
MALVACEAE						
Ceiba aesculifolia	0.06	0.02	Fruit, root	Medicinal	Fruit	156

(Kunth) Britten & Baker f. (Pochote)				Handicrafts	Fruit	
FABACEAE						
Leucaena macrophyla Benth. (E) (Guaje de Hoja Redonda)	0.03	0.02	Seed, leaf sprout	Fuel	Stem, branches	PR
				Pole	Stem	
				Timber	Stem	
MYRTACEAE						
Psidium sartorianum (O. Berg) Nied. (Little guava; Guayabillo)	0.03	0.02	Fruit, leaves	Fuel	Stem, branches	PR
CAPPARACEAE						
Morisonia americana L. (Zapote Barranqueño)	0.03	0.02	Fruit			185
CARICAEAE						
Carica papaya L. (Papayo)	0.03	0.01	Fruit			
CANNABACEAE						
Celtis iguanaea (Jacq.) Sarg. (Iguana hackberry; Granjeno)	0.03	0.01	Fruit			395
SAPOTACEAE						
Pouteria campechiana (Kunth) Baehni (Zapote calentura)	0.03	0	Fruit			PR
LAURACEAE						
Persea americana Mill. (Avocado; Aguacate)	0.03	0	Fruit			
RUTACEAE						
Casimiroa edulis La Llave (White sapote; Zapote Dormilón)	0.03	0	Fruit			PR
Citrus aurantifolia (Christm) Swingle (Key lime; Limón)	0.03	0	Fruit	Timber	Stem	
EBENACEAE						
Diospyros sp. L. (Persimmon tree; Zapotillo Negro)	0.03	0	Fruit			366

LAMIACEAE				
Vitex mollis Kunth (E) (Ahuilote)	0.03	0	Fruit	266
