# Supplementary material for: Multiple Uses of Wild Edible Trees by a Nahua-Origin Community in Western Mexico
Source: Plants (Basel). 2024 Nov 28;13(23):3334. doi: 10.3390/plants13233334 (PMC11644277; doi:10.3390/plants13233334)
Supplement: Supplementary file 1 [file plants-13-03334-s001.zip › Pacheco-Flores et al._Supplementary information_TableS3.pdf]

plants

Table S3. Preparation methods for some WETs. A complete catalog of local recipes is being constructed based on a participatory process, looking for a contribution to the local biocultural memory.

**Dish**	**Ingredients**	**Preparation method**
Guaje verde (*Leucaena leucocephala*) chili sauce	-Guaje seeds<br>-Dried tree chili<br>-Green tomato	-Toast guaje seeds and chili<br>-Roast the tomatoes until cooked<br>-Mash everything in the mortar and add salt to taste.
Scrambled eggs with bonete (*Jacaratia mexicana*)	-Unripe fruits of bonete<br>-Eggs<br>-Chopped onion and tomato	-Cut the ribs off the bonete fruit and let the latex come out. Put the fruits in boiling water for 5 minutes. Remove, peel and cut into squares.<br>-Sauté the onion and tomato, then add the bonete, salt and finally the beaten eggs.
Parota (*Enterolobium cyclocarpum*) seeds with nopales salad	-Unripe parota seeds<br>-Chopped nopales, onion, green chili, coriander and tomato<br>-Lemon juice	-Boil the green parota seed pods and take out the seeds. Remove the whitish seed covering and set aside the rest (green seed).<br>-Boil the nopales. Once cooked, rinse them with clean water and drain.<br>-Mix all the ingredients and add lemon and salt to taste.
Ciruela jam (*Spondias purpurea*)	-Ripe ciruelas<br>-Canela<br>-Sugar	-Place the ripe ciruelas in a pot over low heat and mash them. Add the sugar and cinnamon. Allow to boil, stirring constantly until it becomes thick. It is optional to remove the seeds.
